# Development and Validation of a Diagnostic Algorithm for Down Syndrome Using Birth Certificate and International Classification of Diseases Codes

**DOI:** 10.3390/children11101271

**Published:** 2024-10-21

**Authors:** Lin Ammar, Kristin Bird, Hui Nian, Angela Maxwell-Horn, Rees Lee, Tan Ding, Corinne Riddell, Tebeb Gebretsadik, Brittney Snyder, Tina Hartert, Pingsheng Wu

**Affiliations:** 1 Division of Epidemiology, Vanderbilt University School of Medicine, Nashville, TN 37232, USA; lin.ammar@vanderbilt.edu; 2 Department of Pediatrics, Vanderbilt University Medical Center, Nashville, TN 37232, USA; kristin.m.bird@vumc.org (K.B.); angela.c.maxwell-horn@vumc.org (A.M.-H.); tina.hartert@vumc.org (T.H.); 3 Department of Biostatistics, Vanderbilt University Medical Center, Nashville, TN 37232, USA; hui.nian@vumc.org (H.N.); tan.ding@vumc.org (T.D.); tebeb.gebretsadik@vumc.org (T.G.); 4 Department of Pediatrics, College of Medicine, University of Arizona, Tucson, AZ 85721, USA; reeslee@arizona.edu; 5 Division of Biostatistics and Epidemiology, School of Public Health, University of California, Berkeley, CA 94720, USA; 6 Department of Medicine, Vanderbilt University Medical Center, Nashville, TN 37232, USA; brittney.m.snyder@vumc.org

**Keywords:** Down syndrome, administrative databases, International Classification of Diseases, birth certificate

## Abstract

Objective: We aimed to develop an algorithm that accurately identifies children with Down syndrome (DS) using administrative data. Methods: We identified a cohort of children born between 2000 and 2017, enrolled in the Tennessee Medicaid Program (TennCare), who either had DS coded on their birth certificate or had a diagnosis listed using an International Classification of Diseases (ICD) code (suspected DS), and who received care at Vanderbilt University Medical Center, a comprehensive academic medical center, in the United States. Children with suspected DS were defined as having DS if they had (a) karyotype-confirmed DS indicated on their birth certificate; (b) karyotype-pending DS indicated on their birth certificate (or just DS if test type was not specified) and at least two healthcare encounters for DS during the first 6 years of life; or (c) at least three healthcare encounters for DS, with the first and last encounter separated by at least 30 days, during the first six years of life. The positive predictive value (PPV) of the algorithm and 95% confidence interval (CI) were reported. Results: Of the 411 children with suspected DS, 354 (86.1%) were defined as having DS by the algorithm. According to medical chart review, the algorithm correctly identified 347 children with DS (PPV = 98%, 95%CI: 96.0–99.0%). Of the 57 children the algorithm defined as not having DS, 50 (97.7%, 95%CI: 76.8–93.9%) were confirmed as not having DS by medical chart review. Conclusions: An algorithm that accurately identifies individuals with DS using birth certificate data and/or ICD codes provides a valuable tool to study DS using administrative data.

## 1. Introduction

Down syndrome (DS), characterized by the presence of an extra copy of chromosome 21, is the most common chromosomal abnormality among live-born infants [1]. Individuals with DS are at increased risk for various co-occurring health conditions in the gastrointestinal, neurological, sensory, metabolic, endocrine and respiratory systems, and are at increased risk of mortality compared to those without DS [2,3]. The National Institute of Health Investigation of Co-occurring conditions across the Lifespan to Understand Down syndrome (NIH INCLUDE) project, launched in 2018, calls for the assembly and study of large populations of individuals with DS to follow the development of the condition over time and to fully characterize condition traits at different stages of development [4].

Administrative data provide a time- and cost-saving approach to gather information on a large number of children over time. Individuals of interest can be identified using International Classification of Diseases (ICD) diagnosis codes. Because ICD codes are collected for billing rather than research purposes, ICD-based diagnoses are subject to biases that threaten their clinical validity. Codes that are reimbursed well are more likely to be included in a patient’s record compared with those that are not [5,6]. Further, codes that represent disease conditions that function often as comorbidities rather than the primary reasons for clinical encounters are less likely to show up on an individual’s record, particularly in disease conditions that are defined by vague clinical criteria [7,8,9]. Indeed, a previous study identifying individuals with DS using ICD-9 codes specific to DS observed only moderate sensitivity (87%) and positive predictive value (PPV: 79%) [10].

The objective of this study was to develop an algorithm using birth certificates and/or claims data (ICD codes) to identify individuals with DS and validate the algorithm using manual chart review, a process that examines a patient’s health documentation such as orders, results, reports, and diagnostics. If an accurate algorithm is identified, it can be used to identify individuals with DS for future research studies using administrative data.

## 2. Materials and Methods

### 2.1. Study Design and Cohort

**Figure 1:**
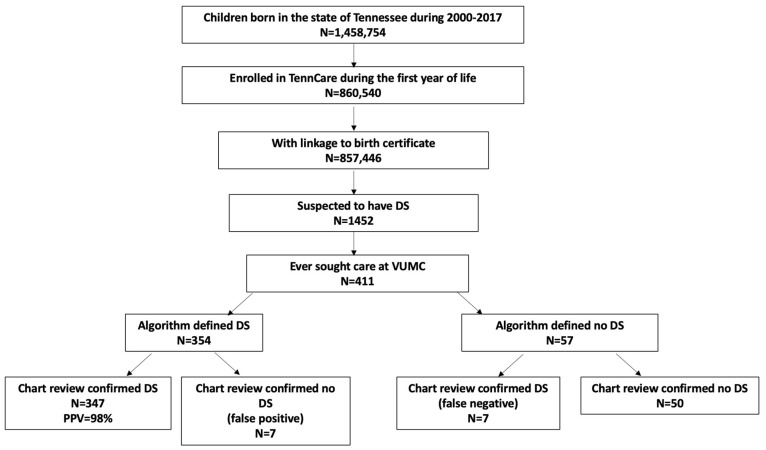
Study cohort identification.

We used data from the Tennessee Medicaid Program, TennCare, which primarily offers healthcare to low-income pregnant women, caretakers of a minor child, children, elderly people and individuals with a disability (https://www.tn.gov/tenncare.html, accessed on 16 October 2024). To qualify for TennCare, an individual must adhere to specific income and resource limits. We identified children born between 2000 and 2017 who were continuously enrolled in TennCare during their first year of life and whose records were linked to their birth certificates. We then identified children suspected of having DS because they either had DS recorded on their birth certificate or they had at least one ICD diagnostic code of DS (ICD-9: 758.0; ICD-10: Q90) within any of the diagnostic fields during healthcare encounters up to age six. Children were followed to age six years, death, disenrollment, or the end of the study (31 December 2020), whichever came first. We restricted this cohort to those who ever sought care at Vanderbilt University Medical Center (VUMC), a comprehensive academic medical center in the United States, in order to perform a manual chart review of their records (n = 411, 28.3%) (Figure 1). The diagnosis of DS was confirmed through a manual review of electronic medical records, utilizing a structured chart abstraction form to ensure consistent data capture across all records. This thorough review served as the gold standard for validating our algorithm. For children with chart-review-confirmed DS, type of DS, timing of first diagnosis (prenatal vs. postnatal), and method of diagnosis were determined. The study protocol was approved by the VUMC and Tennessee Department of Health Institutional Review Boards.

### 2.2. Algorithm Defining DS

We developed an algorithm for DS that used birth certificate data and/or ICD diagnosis codes (ICD-9: 758.0; ICD-10: Q90). A new birth certificate format was adopted in 2004. Prior to 2004, there was a single check box to indicate DS. From 2004 onward, there were separate indications for karyotype-confirmed and karyotype-pending DS.

Children were defined as having DS if they met one of the following criteria:

1. Having birth certificate indication for “karyotype-confirmed” DS;
2. Having birth certificate indication for “karyotype-pending” DS or just DS if test type was not specified (i.e., prior to 2004) and having at least two healthcare encounters for DS during the first six years of life;
3. Having at least three healthcare encounters for DS during the first six years of life, with the first and last encounter separated by at least 30 days.

### 2.3. Statistical Analysis

Demographic characteristics of the study population were described using median (interquartile range: IQR) values for continuous variables and frequency and proportion for categorical variables.

Among individuals whom the algorithm defined as having DS, we calculated the positive predictive value (PPV), the proportion of the study population whose DS was confirmed by manual abstract review. Among individuals whom the algorithm defined as not having DS, we calculated the proportion who did not have DS according to manual abstract review. The corresponding 95% confidence intervals (CIs) were calculated using Wilson’s formula [11]. All analyses were performed using R software version 4.3.1 (R foundation for statistical computing, Vienna, Austria).

## 3. Results

There were 857,446 individuals born between 2000 and 2017 who were continuously enrolled in TennCare during their first year of life and whom we could link to their birth certificates. Of these, 1452 were suspected to have DS by having either an indication for DS on their birth certificate or an ICD diagnosis code for DS during their first six years of life.

Of the 1452 children with suspected DS, 411 ever sought care at VUMC (Figure 1). All 411 children with suspected DS had at least one ICD diagnosis code for DS, and 24.8% (n = 102) had DS coded on their birth certificate (Table 1). These children had predominantly singleton births (98.3%) and older siblings at the time of birth (69.7%). Fifty-five percent of the children were assigned male at birth. The median gestational age was 38 weeks (IQR 36, 39) and the median birthweight was 2920 g (IQR 2495, 3280). A large proportion of these children had congenital heart disease (83.5%) (Table 2).

Among these 411 children with suspected DS, our algorithm determined that 354 (86.1%) had DS by meeting at least one of the criteria (Figure 1). Table 3 presents the detailed number of children determined as having DS by meeting each combination of the criteria defined in the algorithm. Of the 102 children who had DS coded on their birth certificate, 101 (99.0%) were determined as having DS by meeting either criterion 1 or criterion 2. Three hundred and fifty children met criterion 3 (3+ ICD diagnoses for DS criterion). These children composed 98.9% of the population identified by the algorithm.

When compared to the gold standard of manual medical chart review, 347 out of the 354 algorithm-defined DS children had their DS confirmed, for a PPV of 98.0% (95% CI: 96.0–99.0%). Examination of criterion-specific PPV suggested that the 101 children with a birth certificate indication and that met criterion 1 or 2 had DS confirmed by chart review (PPV = 100%). Among the 253 children who exclusively met the 3+ ICD diagnoses for DS criterion (criterion 3), 97.2% (95%CI: 94.4–98.7%) had DS confirmed by chart review (Table 3). Of the 57 children who did not satisfy any of the algorithm’s criteria for DS, 87.7% (95% CI: 76.8–93.9%) were true negatives based on chart review (Figure 1). There were a total of seven children whom the algorithm incorrectly identified as having DS (false positives) and seven children who were incorrectly identified as not having DS (false negatives) (Figure 1, Table 3). The median number of ICD billing codes for DS which appeared in the medical records was 4 (range: 3, 14) and 1 (range: 1, 2) over 6.00 (range 4.00, 6.00) and 4.71 (range 1.23, 5.25) years for the false positives and false negative children, respectively. The corresponding median age when an ICD code for DS first appeared on each child’s billing record was 111 days (range 11, 1673) for the false positives and 97 days (range 0 [at the birth hospitalization], 1079) for the false negative children.

Among children whose DS was confirmed by medical chart review, we further determined the type of DS, diagnosis method, and timing of first DS diagnosis (prenatal vs. postnatal). Of the 283 children whose type of DS was known, 266 (94.0%) had Trisomy 21 nondisjunction (Table 4). Among the 293 children whose timing of first diagnosis was known, 28.3% (n = 83) were diagnosed prior to delivery. Though not statistically significant (trend test *p* = 0.16), the proportion of children whose DS was confirmed prenatally increased over time: 27.5% (born 2000–2004), 23.5% (born 2005–2009), 28.4% (born 2010–2014), and 34.8% (born 2015–2017). Further examination found that 38.6% (n = 32) of children who were diagnosed prior to delivery had DS indicated on the birth certificate. Of the 246 DS children whose diagnosis method was known, 92.3% of subjects had karyotype and 8.1% had fluorescence in situ hybridization (Table 4). About 4% of children (n = 13) had non-invasive prenatal testing for DS. A close examination of the 57 children whose chart review confirmed no DS suggested that the majority of them had or were suspected to have some sort of chromosomal abnormality (73.4%) and had congenital heart disease (73.7%).

## 4. Discussion

Using birth certificate data and ICD diagnosis data, we developed an algorithm to accurately determine children with DS. Of the 411 children who were suspected to have DS and sought care at VUMC, our algorithm defined 86.1% as having DS. Manual chart review confirmed that the algorithm is accurate in differentiating suspected DS children with and without DS, with 98.0% and 87.3% accuracy, respectively, in determining those truly with DS and those truly without DS.

We found that the birth certificate as a source in DS case identification was less sensitive. Only approximately one-quarter of children with suspected DS had the condition coded on their birth certificate. The birth certificate is typically filled out 24 to 48 h after birth, making a postnatal diagnosis of DS unlikely to be fully confirmed at the time of record filling [12]. Among children whose condition was known prior to delivery, less than 40% had DS coded on their birth certificate. This may reflect a more careful practice allowing physicians to have a questioning attitude if testing was performed with a maternal blood test or only mild DS signs were noted. On the other hand, in this cohort of children with suspected DS, a DS indication on the birth certificate had high predictive value. In our study, only 1 out of 102 subjects had an incorrect indication on their birth certificate. By requesting either karyotype-confirmed DS (criterion 1) or two more ICD-coded visits in addition to karyotype-pending DS (or simply DS if no test indicated) (criterion 2), our algorithm had correctly identified all 101 children whose DS was confirmed by chart review (PPV = 100%). It is important for future research to be aware of the strength and limitations of using birth certificate indication in DS case identification.

Our finding that not all children with an ICD code for DS truly had DS is consistent with the previous literature and highlights that there are some inaccuracies within the administrative billing data such that billing codes can be inappropriately assigned to patient encounters [10]. The majority of children with an inappropriate indication and/or billing(s) for DS have or were suspected to have a different chromosomal abnormality. It is important to note that consistent wrong indications for DS are not common in practice. By requiring repeated coding across independent healthcare encounters, our algorithm significantly reduced misclassification. We further required a minimum one-month interval between the first and last ICD-coded encounters, when ICD coding was used alone, to ensure there were at least two independent healthcare visits that were likely for different health issues/episodes with codes for DS.

An important contribution is the finding that ICD coding alone can be an efficient and valid approach in DS case identification. Almost all children our algorithm defined as having DS satisfied the 3+ ICD coding criteria and overwhelmingly were confirmed to have DS by chart review. As birth certificate data are not always accessible in research settings that rely on administrative and electronic medical record data, an algorithm based on ICD coding criteria enables broad application.

Despite the widespread availability of prenatal testing for DS, only 28% of children with DS were identified prenatally over the study period. The proportion with prenatal diagnosis among live-born children with DS increased slightly over time. As our study included children who were alive until at least age one (per study inclusion criteria of continuous enrollment during the first year of life), the low proportion of children with DS whose condition was diagnosed prenatally may not directly reflect the overall prenatal testing rate, as diagnoses resulting in pregnancy terminations and infant death are not included in the study population. In a systematic review, it was estimated that 67% to 85% of individuals with a positive prenatal diagnosis of DS terminate their pregnancy in the US [13]. Second, approximately 5% of infants born with DS die during infancy and thus were not be included in our study population [14].

Our study has several limitations. We developed and applied our algorithm to a study population of children who have regular and frequent healthcare visits. DS as a comorbidity may be more likely to be coded during well-child visits in children with DS with fewer comorbidities than among children with DS with significant comorbidities and specialty care and more frequent disease-driven clinical encounters. Requirement of at least three independent indications/visits for DS could overlook children with DS with fewer comorbidities and requirements for clinical care. This would be particularly true when children were followed within a limited time period. Of the seven children with chart-review-confirmed DS whom the algorithm failed to identify, the median follow-up time was 4.71 years, 1.28 years shorter than that of children whom the algorithm defined as having DS. We also restricted our study population to those who sought care at a single academic medical center, which may not be generalizable in other settings. Indeed, a comparison of a similar cohort of children born and enrolled in Medicaid in the same time period, but who never sought care at the medical center (i.e., 1452 − 411= 1041, Figure 1), suggested that although all of them had at least one healthcare visit with ICD codes for DS, children with suspected DS who never sought care at VUMC were less likely to have an indication of DS on their birth certificate and less likely to be defined as having DS by the algorithm (*p* < 0.001) (Table S1). Children with suspected DS who sought care at VUMC were more likely to have congenital heart disease and had older, more educated mothers. These mothers were more likely to be married and less likely to smoke during pregnancy (Table S2). Lastly, our study population of children with suspected DS had, by design, a higher likelihood of DS than the general population. When applied to a general population, the algorithm may not perform as well. The algorithm may not identify children with DS if they have limited healthcare encounters and/or have healthcare encounters not frequently coded for DS.

## 5. Conclusions

In conclusion, we developed an algorithm that used data from birth certificates and ICD codes and had a high PPV for identifying children with DS. Application of this algorithm will provide a reliable and efficient approach to identify and study individuals with DS, and the algorithm remains accurate for researchers who do not have access to birth certificate data and use ICD codes alone.

## Figures and Tables

**Table 1:** Children who were suspected of having DS by an indication for DS on the birth certificate and/or with at least one ICD diagnosis for DS, born in 2000–2017, enrolled in TennCare during infancy, and ever sought care at VUMC.

Qualified for Suspected DS	Confirmed DS		Total (n = 411)
	Yes (n = 354)	No (n = 57)	
Having at least one ICD code specific for DS	354 (100%)	57 (100%)	411 (100%)
DS coded on birth certificate	101 (28.5%)	1 (<1%)	102 (24.8%)

**Table 2:** Maternal and infant characteristics of children with suspected DS (an indication for DS on the birth certificate and/or with at least one ICD diagnosis for DS), who were born in 2000–2017, enrolled in TennCare during infancy, and ever sought care at VUMC.

	Children with suspected DS (n = 411)
Maternal characteristics	
Age at delivery, median (25th percentile, 75th percentile)	31 (23, 38)
Education (n = 410)	
Some high school or less	108 (26.3%)
High school graduate	133 (32.4%)
At least some college education	169 (41.2%)
Residence	
Urban	134 (32.6%)
Suburban	136 (33.1%)
Rural	141 (34.3%)
Married	241 (58.6%)
Smoking during pregnancy (n = 409)	72 (17.6%)
Prenatal care started at first trimester (n = 381)	264 (69.3%)
Parity (n = 405)	
Primiparous	123 (30.4%)
2	116 (28.6%)
3+	166 (40.9%)
Delivery method	
Vaginal/assisted	217 (52.8%)
Cesarean section	194 (47.2%)
Infant characteristics	
Sex	
Male	224 (54.5%)
Female	187 (45.5%)
Race and Ethnicity	
Non-Hispanic White	209 (50.9%)
Non-Hispanic Black	-- ^1^
Hispanic	-- ^1^
Other	-- ^1^
Gestational age in weeks, median (25th percentile, 75th percentile)	38 (36, 39)
Birth weight in grams, median (25th percentile, 75th percentile)	2920 (2495, 3280)
Small for gestational age at 10th percentile (n = 410)	59 (14.4%)
Singleton birth	404 (98.3%)
One or more older siblings (n = 406)	283 (69.7%)
Congenital heart disease	343 (83.5%)
Birth year	
2000–2004	74 (18.0%)
2005–2009	118 (28.7%)
2010–2017	219 (53.3%)

^1^ Cells were suppressed for n < 11.

**Table 3:** Algorithm criterion-specific determination of DS and the corresponding positive predictive value among children suspected of having DS who were born in 2000–2017, enrolled in TennCare during infancy, and ever sought care at VUMC.

Criterion 1 (Karyotype-Confirmed DS)	Criterion 2 (Karyotype-Pending DS or Just DS and ≥2 ICD Diagnosis for DS)	Criterion 3 (≥3 ICD Diagnosis for DS)	Study Population—Children with Suspected DS (n = 411)	Children with Chart-Review-Confirmed DS (n = 354)	Children with Chart-Review-Confirmed No DS (n = 57)	PPV
X ^3^			1	1	0	100.0%
X ^3^		X ^3^	34	34	0	100.0%
	X ^3^		3	3	0	100.0%
	X ^3^	X ^3^	63	63	0	100.0%
		X ^3^	253	246	7 ^1^	97.2%
			57	7 ^2^	50	NA ^4^

^1^ Seven children who had DS identified by the algorithm that was not confirmed by chart review (false positives). ^2^ Seven children who were identified as not having DS by the algorithm that was confirmed by chart review (false negatives). ^3^ X indicates that the corresponding cell criteria was met. ^4^ PPV could not be calculated for individuals that did not meet any of the algorithm defining DS criteria.

**Table 4:** Type, method, and timing of DS first diagnosis among children with DS confirmed by medical chart review (n = 354).

	N (%)
Type (n = 283 ^1^)	
Nondisjunction	266 (94.0%)
Translocation/Mosaic	17 (6.0%)
Diagnosis/confirmation method ^2^ (n = 246 ^1^)	
Karyotype	227 (92.3%)
Fluorescence in situ hybridization	20 (8.1%)
Timing of first diagnosis (n = 293 ^1^)	
Prenatal	83 (28.3%)
Postnatal	210 (71.7%)
Infant age in days when first DS-specific ICD diagnosis appeared in claims data	0 (0, 40) ^3^

^1^ There are 71, 108, and 61 children, respectively, whose type, method, and timing of DS first diagnosis are unknown in the medical charts. ^2^ Not mutually exclusive. ^3^ Median (interquartile range) of 0 days corresponds to a diagnosis made during the birth hospitalization.

## Data Availability

The data to support the findings of this study were from the Division of TennCare in the Tennessee Department of Finance and Administration and from medical records for healthcare encounters at Vanderbilt University Medical Center. De-identified data are available on request from the corresponding author, with the approvals from TennCare and VUMC Institutional Review Board. Data are not publicly available due to privacy concerns and regulatory compliance.

## Supplementary Materials

The following supporting information can be downloaded at https://www.mdpi.com/article/10.3390/children11101271/s1: Table S1: Children who were suspected of having DS by an indication for DS on the birth certificate and/or with at least one ICD diagnosis for DS, born 2000–2017, enrolled in TennCare during infancy, and ever/never sought care at VUMC; Table S2: Maternal and infant characteristics of children with suspected DS, born 2000–2017, enrolled in TennCare during infancy, and ever/never sought care at VUMC.
