# Supplementary material for: Development and Validation of a Diagnostic Algorithm for Down Syndrome Using Birth Certificate and International Classification of Diseases Codes
Source: Children (Basel). 2024 Oct 21;11(10):1271. doi: 10.3390/children11101271 (PMC11506645; doi:10.3390/children11101271)
Supplement: Supplementary file 1 [file children-11-01271-s001.zip › children-3212829-supplementary.pdf]

Development and Validation of a Diagnostic Algorithm for Down Syndrome Using Birth Certificate and International Classification of Diseases Codes

Lin Ammar, Kristin Bird, Hui Nian, Angela Maxwell-Horn, Rees Lee, Tan Ding,

Corinne Riddell, Tebeb Gebretsadik, Brittney Snyder, Tina Hartert, Pingsheng Wu

Table S1: Children who were suspected of having DS by an indication for DS on the birth certificate and/or with at least one ICD diagnosis for DS, born 2000-2017, enrolled in TennCare during infancy, and ever/never sought care at VUMC.

	Ever sought care at VUMC		P value
	Yes (N=411)	No (N=1041)	
Qualified for suspected DS			
Having at least one ICD code specific for DS	411 (100%)	1041 (100%)	1
DS coded on birth certificate	102 (24.8%)	162 (15.6%)	<0.001
Algorithm defined as having DS	354 (86.1%)	726 (69.7%)	<0.001^
Criterion* 1 only	1 (0.2%)	4 (0.4%)	
Criterion 1 & 3 only	34 (8.3%)	38 (3.7%)	
Criterion 2 only	3 (0.7%)	1 (0.1%)	
Criterion 2 & 3 only	63 (15.3%)	113 (10.9%)	
Criterion 3 only	253 (61.6%)	570 (54.8%)	
Algorithm defined as *not* having DS	57 (13.9%)	315 (30.3%)	

*Criterion 1: Karyotype confirmed DS; Criterion 2: Karyotype pending DS + 2 ICD coded visits; Criterion 3: 3 ICD coded visits with first and last visits separately by at least 30 days apart.

^P value comparing algorithm defined DS and not DS by ever/never sought care at VUMC

Table S2: Maternal and infant characteristics of children with suspected DS, born 2000-2017, enrolled in TennCare during infancy, and ever/never sought care at VUMC.

	Ever sought care at VUMC		P value
	Yes (411)	No (1041)	
**Maternal characteristics**			
Age at delivery (n=1450)	31 (23, 38)	27 (22, 36)	<0.001
Education (n=1446)			0.007
Some high school or less	108 (26.3%)	305 (29.4%)	
High school graduate	133 (32.4%)	394 (38.0%)	
At least some college education	169 (41.2%)	337 (32.5%)	
Residence (n=1451)			<0.001
Urban	134 (32.6%)	279 (26.8%)	
Suburban	136 (33.1%)	249 (23.9%)	
Rural	141 (34.3%)	512 (49.2%)	
Married (n=1451)	241 (58.6%)	456 (43.8%)	<0.001
Maternal smoking during pregnancy (n=1446)	72 (17.6%)	236 (22.8%)	0.03
Prenatal care started at first trimester (n=1341)	264 (69.3%)	679 (70.7%)	0.60
Parity (n=1441)			0.61
Primiparous	123 (30.4%)	341 (32.9%)	
2	116 (28.6%)	277 (26.7%)	
3+	166 (40.9%)	418 (40.3%)	
Delivery method			0.03
Vaginal/assisted	217 (52.8%)	617 (59.3%)	
Cesarean section	194 (47.2%)	424 (40.7%)	
**Infant characteristics**			
Male sex	224 (54.5%)	586 (54.7%)	0.80
Race and Ethnicity			
Non-Hispanic White	209 (50.9%)	430 (41.3%)	<0.001
Non-Hispanic Black	--*	186 (17.9%)	
Hispanic	--	153 (14.7%)	
Other	--	272 (26.1%)	

Gestational age in weeks (n=1448)	38 (36, 39)	38 (36, 39)	0.26
Birth weight in grams	2920 (2495, 3280)	2892 (2410, 3260)	0.15
Small-for-gestational-age at 10th percentile (n=1446)	59 (14.4%)	151 (14.6%)	0.93
Singleton birth	404 (98.3%)	1014 (97.4%)	0.31
One or more older siblings (n=1441)	283 (69.7%)	694 (67.1%)	0.33
Congenital heart disease	343 (83.5%)	689 (66.2%)	<0.001
Birth year			
2000-2004	74 (18.0%)	295 (28.3%)	<0.001
2005-2009	118 (28.7%)	301 (28.9%)	
2010-2017	219 (53.3%)	445 (42.7%)	

*Cells were suppressed for n<11
